# MicroRNAs in Kidney Transplantation: Living up to Their Expectations?

**DOI:** 10.1155/2015/354826

**Published:** 2015-05-11

**Authors:** Eline K. van den Akker, Frank J. M. F. Dor, Jan N. M. IJzermans, Ron W. F. de Bruin

**Affiliations:** Department of Surgery, Erasmus MC University Medical Center, P.O. Box 2040, 3000 CA Rotterdam, Netherlands

## Abstract

Since the discovery of microRNAs, ample research has been conducted to elucidate their involvement in an array of (patho)physiological conditions. Ischemia reperfusion injury is a major problem in kidney transplantation and its mechanism is still not fully known, nor is there an effective therapy. Furthermore, no biomarker is available to specifically measure (ischemic) damage after kidney transplantation or predict transplantation outcome. In this review, we summarize studies conducted on microRNAs in renal ischemia reperfusion injury and kidney transplantation. Although the number of publications on miRNAs in different areas of nephrology is increasing every year, only a limited number of reports that address the role of miRNAs in relation to ischemia reperfusion injury or kidney transplantation are available. All reports up to June 2014 on microRNAs in renal IRI, kidney transplantation, and renal allograft status were included. Design of the studies was highly variable and there was limited overlap between microRNAs found in these reports. No single microRNA expression pattern could be found, although multiple microRNAs involved in the immune response seem to be altered after ischemia reperfusion injury and kidney transplantation. Although there is a growing interest in microRNA research in kidney transplantation aiming to identify biomarkers and therapeutical targets, to date, no specific microRNA has been demonstrated to be applicable as either one, mostly because of lack of specificity. More systematical research is needed to determine whether microRNAs can be applied as biomarker, therapeutic target, or therapeutic agent in kidney transplantation.

## 1. Introduction

Kidney transplantation (KT) is the treatment of choice for end stage renal failure. Ischemia reperfusion injury (IRI) is an inevitable consequence of KT. Ischemia leads to deprivation of nutrients and oxygen resulting in ATP depletion, loss of ion gradients, cell swelling, and increase of toxic by-products. Although contradictive, reperfusion enhances the damage by flow of oxygen rich blood, production of oxygen-free radicals, and activation of an inflammatory response [1]. After KT, IRI may induce delayed graft function (DGF), which is associated with increased comorbidity and longer hospital stay and is associated with acute and chronic rejection [2]. Despite extensive research, the specific mechanisms behind IRI are still not fully understood and no specific therapy is available. Preventing or treating IRI at an early stage could prevent DGF and thereby contribute to fast patient recovery, shortened hospital stay, and improved graft function.

MicroRNAs (miRNAs) are small, ~19–25 nucleotide long, single-stranded RNA molecules which play an important role in posttranscriptional regulation of gene expression by inhibiting translation of target mRNAs. miRNAs are estimated to regulate approximately 60% of all transcripts. Let-7 and lin-4 were the first miRNAs discovered in* Caenorhabditis elegans* in 1993 by Lee and Ambros [3]. Five years later Fire and Mello reported on double-stranded RNA (dsRNA) that could silence genes through RNA interference (RNAi) [4]. Since then, miRNAs have been found in a wide variety of species from plants to viruses and up till now several hundred types are known in humans [3]. Other types of small noncoding RNAs have been found in plants and animals, like small-interfering RNAs and Piwi-interacting RNAs (piRNAs). In this review, we will focus mainly on miRNAs. Their small size and their stability and presence in body fluids make them promising candidates, both as therapeutical targets and as biomarkers. In the last years, much progress has been made in the understanding of the role of miRNAs in (patho)physiologic mechanisms. The goal of this review is to summarize the current knowledge of the role of miRNAs in transplant related IRI and kidney transplantation, with emphasis on their mechanistic role and use as biomarker and as either therapeutic target or agent.

## 2. Methods

We performed a PubMed-search containing the MeSH-terms “microRNA” and “renal ischemia reperfusion injury.” All abstracts were read and only those reports were selected when the main topic was microRNA-expression after renal ischemia reperfusion injury. We performed another search with “microRNA” and “kidney transplantation.” Again only those reports were selected where the main topic was microRNA-expression and after kidney transplantation. Non-English papers were excluded. One report on microRNA and ischemia reperfusion injury was focused on a new method of analysis and was therefore excluded.

### 2.1. miRNAs in the Kidney

#### 2.1.1. Tissue Specificity

As miRNAs play an important role in the regulation of protein synthesis, measurement of their expression may provide information about the physiology of organs in normal and diseased states. Since organs have a different spectrum of miRNA-expression, it was suggested that tissues contained specific miRNAs. However, in an expression atlas of human and mouse miRNAs, great similarity between the two species was found, and exclusive expression in an organ appeared to be very rare [5]. Most miRNAs were found in more than one organ. Expression of most miRNAs was ubiquitous, and some showed some degree of tissue specificity. miR-16 was found in all tissues and demonstrated to be the most abundant miRNA. miR-21 was also detected in most tissues, but abundance varied. Expression was higher in malignant cell lines. It was found that miRNAs that were previously considered as tissue specific were actually present in low concentrations in other tissue types as well [6]. Therefore, it was suggested to redefine tissue specific miRNAs as preferentially expressed or organ-enriched miRNAs.

Several organ-enriched miRNAs were found in the human kidney; miRNA-192, miRNA-194, miRNA-204, miRNA-215, and miRNA-216 were more abundant in kidney as compared to heart, lung, spleen, muscle, and prostate [7].

In the rat kidney, different expression profiles between the cortex and medulla have been described [8]. miRNA-192 and miRNA-194 were significantly more abundant in the cortex. Eleven miRNAs were found with a higher expression in the medulla, among them miR-30c and miR-200c.

#### 2.1.2. miRNAs in Ischemia Reperfusion Injury and Kidney Transplantation

Studies on miRNAs in renal IRI and KT have mainly focused on finding an early and specific biomarker for graft function. Serum creatinine is now used to monitor the function of the graft but is a late biomarker with low specificity and sensitivity. Seven studies have used human kidney biopsies to study miRNA expression in normal grafts compared to those with acute rejection or interstitial fibrosis and tubular atrophy (IF/TA) [9–14]. One study focused on expression differences between operationally tolerant patients and patients with stable graft function under immunosuppression [15]. In five studies, animal experiments were used to investigate the involvement of miRNAs in renal IRI [16–20]. An overview is given in Table 1. Most studies first perform genome-wide expression profiling using kidney tissue, either after renal IRI in animals or from clinical biopsies or urine samples. To verify expression levels of a miRNA of interest, qRT-PCR is used. To further investigate their function, the majority of the reports use kidney cell lines such as HK-2, HRC, or PTEC, or PBMCs.

### 2.2. *In Vivo* Studies

Dicer is an enzyme found in the cytosol and cleaves precursor miRNA to produce mature miRNAs [21]. Deletion of Dicer is used to study the role of miRNAs in different organ systems. Mice with targeted deletion of Dicer in renal proximal tubule develop normally with no abnormalities in the kidney [16]. Under normal conditions 80% of 173 measured miRNAs in these mice had a significantly lower expression in the kidney compared to wild type controls. After bilateral renal ischemia for 30 minutes, these mice were protected against IRI. Survival was significantly higher in the Dicer-knockout group, and damage to the proximal tubule decreased. miRNA-profiling in kidney after 12 and 48 hours of reperfusion showed several up- and downregulated miRNAs in the cortex (with a log⁡2 fold change >2) (Table 1). Of the 13 upregulated miRNAs, only two (miR-132, miR-362) were both upregulated after 12 and after 48 hours. In the downregulated miRNAs only one (miR-379) was found at both time points. Both upregulated miR-132 and downregulated miR-379 are thought to play a role in the MAPK-pathway, which is known to be involved in renal IRI [22, 23].

To study the effect of lymphocyte infiltration on miRNA expression, R°/c*γ*°mice that lack NK cells, NKT cells, B cells, and T cells were subjected to 30 minutes of renal IRI, and miRNA expression was compared to wild type mice [17]. Differential expression of miR-21, miR-20a, miR-146a, miR-199a-3p, miR-214, miR-192, miR-187, miR-805, and miR-194 in the kidney of both groups was found. The miRNA expression pattern over a time course after IRI was similar between wild type and R°/c*γ*°mice, suggesting that the miRNA profile after IRI seems to be independent of infiltrating lymphocytes.

When IRI was compared to sham-operated mice, 5 differentially expressed miRNAs were identified in the kidney [24]. Three of them, miR-17-5, miR-21, and miR-106, had fold changes of more than 30% at 24 hours after reperfusion. miR-17-5 and miR-106 belong to the same miR-17 family and had similar expression patterns. In a time course, it was seen that miR-17-5 was upregulated from 24 hours until 3 days after reperfusion. miR-21 was upregulated at 3 and 4 days after reperfusion. At all measured time points (1, 3, and 4 days after reperfusion), a significant correlation was found between miR-17-5 and miR-21. The time points at which these miRNAs were upregulated may give some insight in which mechanism they are involved, either the injury and maintenance phase (miR-17-5) or recovery phase (miR-21). Their correlation suggests that these miRNAs influence each other's expression.

To study miRNA profiles after renal IRI in rats, renal pedicles were clamped for 30 minutes and miRNA expression in kidney, blood, and urine was compared to sham-operated rats at 24, 72, and 120 hours after reperfusion [18]. A >2-fold difference in expression was considered significant. Among the miRNAs which were differentially expressed at all three time points, three miRNAs of interest were further investigated: miR-18a, miR-21, and miR-155. Expression data were validated using qRT-PCR and compared between time points but also between cortex and medulla. miR-21 expression in the cortex increased after reperfusion and remained stable during the time course. In the medulla however expression continued to rise from a fold change of ~2.5 at 24 hours to a fold change of ~13 at 120 hours. miR-155 had a high expression (~6-fold change) at 24 hours in the cortex, which decreased to a fold change of ~3 at 120 hours. In the medulla, expression showed a reversed course, with low expression at 24 hours and a fold change of ~5 at 120 hours.

The course of miR-18a did not differ between cortex and medulla. Furthermore, expression of these miRNAs did not change in other tissues (heart, liver, lung, and spleen) after renal IRI. Expression in whole blood showed a significant decrease of the three miRNAs after reperfusion. In urine, only miR-21 showed a significant increase at 72 hours. miR-18a was not detectable in urine. Using Target Scan, 29 mRNA targets involved in apoptosis and cell proliferation were found for the three miRNAs, but their precise role was not investigated any further.

Ischemic Preconditioning (IPC) has been shown in some models to be an efficient technique to ameliorate damage by IRI in different organs like heart, brain, liver, and kidney [25–27]. The possible involvement of miR-21 in its protection against IRI was studied in a rat model [19]. miR-21 was chosen because of its proapoptotic targets. miR-21 expression was significantly higher after IPC and IRI compared to sham treatment. This corresponded with lower serum creatinine levels at 24 hours after IRI. When anti-miR-21 was administered before IPC, no rise in miR-21 was seen, and protection by IPC was abolished. When anti-miR-21 was administered before IRI without IPC, no effect on the severity of IRI was seen. This suggests that miR-21 is involved in induction of the protective effect of IPC and that administration of miR-21 before IRI may mimic the effects of IPC. These authors also showed that preconditioning with xenon provides similar effects on miR-21 expression as IPC [28]. Pretreatment with xenon, a noble gas with volatile anaesthetic properties, resulted in protection against IRI. Again, this effect was dependent on miR-21 and protection was lost when anti-miR-21 was administered.

Microvesicles derived from endothelial progenitor cells have the ability to induce resistance against renal IRI [20]. This was not seen after administering microvesicles from fibroblasts. When treated with endothelial microvesicles, creatinine and BUN levels were significantly lower compared to untreated controls. Via microarray analysis, 26 miRNAs were found to be expressed in these microvesicles. miR-126 and miR-296 were chosen for further analysis because of their proangiogenic and antiapoptotic effects. Using qRT-PCR, it was found that miR-126 and miR-296 were expressed in endothelial progenitor microvesicles, but not in microvesicles derived from fibroblasts. When anti-miR-126 and anti-miR-296 were added to the microvesicles, the protective effect was lost, showing that the protective effect was dependent on miR-126 and miR-296.

Because the aims, models, methods, and time points used in these studies differ considerably, it is difficult to draw an overall conclusion on the role and relevance of specific miRNAs in IRI. miRNA expression associated with protection against IRI cannot be compared to the profiled miRNAs associated with IRI itself. In the former the emphasis is to find protective miRNAs, while in the latter the pathophysiological response of miRNAs after IRI is measured.

Another reason for the lack in overlap between miRNAs is that initial profiling experiments later randomly select particular miRNAs of interest and neglect the role of other miRNAs found in these experiments. Dicer−/− mice which are protected against renal IRI have a low expression of miR-194. Godwin et al. [17] found a downregulation of miR-194 after renal IRI, which suggests that low expression of miR-194 is involved in protection against IRI (Table 1).

### 2.3. Clinical Studies

Already in 2007, a miRNA profile in patients with acute rejection was reported [9]. Cortical biopsies from three kidneys with acute rejection after live donor KT were compared to 3 biopsies retrieved from kidney tissue after nephrectomy because of a renal tumour. Biopsies were taken on clinical indication. Results showed 8 upregulated and 12 downregulated miRNAs, with a ratio (rejection/normal) <0.5 or >2.0 considered significant (Table 1). Another profiling study compared miRNA expression in biopsies suffering from DGF, acute rejection, or antibody-mediated rejection (AMR) with normal allograft biopsies [14]. In the DGF group, all upregulated miRNAs (Table 1) are involved in cell death and proliferation. Only one miRNA was also upregulated in AMR, miR-182, which was found to be upregulated in a murine IRI model as well [16]. miR-182 inhibits FOXO1, which in turn causes proliferation of T-helper cells and is associated with cardiac allograft rejection in mice and ischemic damage in the brain [16, 29, 30]. Furthermore, an overlap of six miRNAs was found (miR-155, miR-125a, miR-30c, miR-27b, miR-193b, and miR-125b) in the acute rejection set of another study, in which biopsies of 17 normal allografts were compared with 12 allografts with acute rejection [10]. In this study, miR-142-5p, miR-155, and miR-223 were the most significantly upregulated miRNAs in acutely rejected kidneys (*P* < 0.0001). A positive correlation between the expression of the T-cell marker CD3 and miR-142-5p was found. Since miR-142-5p is mostly expressed in hematopoietic cells, its presence can be attributed to the influx of inflammatory cells in the kidney [31]. In PBMCs, expression levels of miR-142-5p may vary and are significantly increased in chronic antibody-mediated rejection compared to stable graft function but are not altered during acute rejection [32]. This suggests that miRNA-142-5p in PBMC may be a marker for antibody-mediated chronic rejection.

Since biopsies are invasive and bear a high risk for complications, a noninvasive method to measure rejection is preferred. miRNAs can be measured in the urine, which could be a promising way to find biomarkers for acute rejection. As mentioned before, miR-21 and miR-155 can be found in rat urine samples. In the same study, urine samples of 22 patients with acute kidney injury admitted to the ICU with high serum creatinine levels and elevated levels of KIM-1 in the urine were collected and miRNA expression was compared to urine samples of 25 healthy volunteers [18]. Among the 22 ICU-patients, 9 were KT-recipients, and 13 had acute kidney injury of their native kidneys. In the patients with AKI (both native and transplant kidneys) an increased expression of miR-21 and decreased expression of miR-155 were found compared to healthy individuals.

Lorenzen et al. compared 68 urine samples of 62 patients with biopsy proven acute rejection with 20 samples of 19 patients with stable graft function [11]. As a disease control group, 13 urine samples of stable transplant recipients with a urinary tract infection were included. Twenty-one miRNAs were found with a >10-fold difference in expression between patients with acute rejection and stable controls. An increase of miR-10a and a decrease of miR-10b and miR-210 were found in urinary samples from patients with acute rejection. miR-210 correlated with glomerular filtration rate over time and a normalization of miR-210 levels was seen after successful antirejection therapy. Compared to the expression values in urinary samples with urinary tract infection only miR-210 showed a differential expression, suggesting miR-210 could act as a urinary biomarker and predictor for acute rejection and long term graft function, respectively.

A similar study was done to find a urinary biomarker for IF/TA [13]. Thirteen biopsies with chronic allograft dysfunction and IF/TA were compared to 5 normal biopsies, all from recipients of a kidney from a deceased donor. 56 differentially expressed miRNAs were found. With use of a target prediction program, fold change, and statistical significance they focused on 5 miRNAs (miR-142-3p, miR-204, miR-107, miR-211, and miR-32) for further research. In urinary samples, a significant difference was found for three of the five miRNAs, namely, miR-142-3p, miR-204, and miR-211. Although the predictive value still has to be established, this miRNA signature is a possible biomarker for IF/TA. With an* in silico* model, targets of these miRNAs were predicted and were found to be the costimulatory molecule CD28 on T-helper cells, B-cell development, and allograft rejection signalling. Other biological functions regulated by this miRNA signature are apoptosis, lymphocyte proliferation, and differentiation of natural killer (NK-) cells, B-cells, and T-cells. Furthermore, a decrease in miR-204 is associated with increased apoptosis in HeLa cells [33]. Using sequencing to profile miRNA expression, biopsies with IF/TA and 4 normal biopsies were compared [12]. In biopsies with IF/TA a differential expression (>2-fold) of several miRNAs (Table 1) was found, including miR-223 which was increased and the miR-30 family, which was decreased. Another study compared IF/TA urine samples to stable graft urine samples. They found 22 differentially expressed miRNAs (*P* < 0.01 with a false discovery rate <15%) (Table 1). Differential expression of miR-99a, miR-140-3p, miR-200b, and miR-200^∗^ was seen both early and late after transplantation (3.73 ± 1.30 and 20 ± 4 months, resp.) [34].

The limited number of human studies that has been conducted so far has yielded hardly any overlap in miRNAs. Comparison of these studies is limited by the use of different patient populations and tissues, like biopsies from cortex, percutaneous core needle biopsies, or urine. Other limitations are the different time points when samples were collected, even within one study. In addition, the focus of the studies differed between acute rejection and IF/TA in which involvement of miRNA undoubtedly differs.

A few miRNAs are found in multiple reports. miR-142-3p is upregulated in both reports on acute rejection [10] and IF/TA [12, 13]. miR-142-3p is mostly found in hematopoietic cells [5] and is associated with upregulation of CD25+ CD4 T-cells [35] and downregulation of heat shock protein (HSP)70 [36]. Another overlapping miRNA, miR-223, is also associated with inflammation, regarded as modulator of neutrophil maturation and fine tuner of granulocyte function [37] and is also found in monocytes [31].

Low expression of the miR-30 family was reported in two experiments. The miR-30 family is shown to be needed in kidney development [38] and miR-30 suppresses apoptosis by targeting the mitochondrial fission machinery [39] (Table 2).

### 2.4. *In Vitro* Studies

In mice, miR-21 was found to play a role in renal IRI [17]. Since miR-21 is associated with cell death, proliferation, and fibrosis [40–42], the role of miR-21 in proliferation and renal fibrosis was studied using primary cultures from murine tubular epithelial cells. Knockdown of miR-21 led to increased cell death, whereas overexpression resulted in decreased cell death. Overexpression of miR-21 did not prevent cell death in cells undergoing ischemia, however.

HIF-1*α* is protective against IRI as it promotes alterations in expression of genes involved in tissue repair after ischemia. miRNA-127 was found to be involved in the HIF-1*α* pathway, and IRI in NRK52E rat kidney cells and HK-2 cells stabilized HIF-1*α* expression, which upregulated miR-127. Following renal ischemia and reperfusion in rats, an increased expression of rno-miR-127 was found, and Kinesin Family Member 3B (KIF3B), which is involved in cellular endocytosis, was identified as a novel target of miR-127 [43].

In human PBMCs from recipients with stable graft function under immunosuppression, expression of miR-106b, miR-142-3p, miR-450b-5p, and miR-876-3p was significantly lower and miR-98, miR-148b, miR-324-5p, and miR-508-3p were significantly higher expressed, compared to operationally tolerant recipients [15].

When PBMCs of healthy volunteers were stimulated with phytohemagglutinin A (PHA) and IL-2, expression of miR-142-3p, miR-324-5p, and miR-450b-5p decreased and expression of miR-876-3p increased. Further investigation showed higher expression of miR-142-3p in B-cells in operationally tolerant patients.

PHA stimulation of PBMCs was also performed to study the presence of miRNAs found earlier in human kidney biopsies [10]. Of the 7 upregulated miRNAs in the kidney, only miR-155 was increased in stimulated PBMCs, whereas let-7c was decreased. miR-223 and miR-142-5p, however, were decreased in PBMCs in contrast to their upregulation in kidney tissue.

In HK-2 cells, it was found that IRI causes a significant downregulation of miR-205. IRI was induced by culturing cells in low oxygen (0.1%) for 16 hours, followed by 3 or 10 hours of reoxygenation [44]. Lower expression of miR-205 was associated with a decreased cell survival. In cells overexpressing miR-205, there was a significant better cell survival after IRI. This protection was associated with the suppression of EGLN2 and an increase of HIF-1*α* and HO-1.

One* in vitro* model focused on IF/TA induced by hypoxia in renal epithelial cells. The authors found downregulation of miR-124 after 48 hours of hypoxia. miR-124 seems to regulate MMP-2, a fibrosis associated gene, and overexpression of miR-124 reversed increased proliferation after hypoxia, although this was independent of MMP-2 [45] (Table 3).

Well-recognized limitations of these* in vitro* studies are that the pathology of IRI is difficult to capture* in vitro*, [19] that mostly only one cell line is studied and the focus is on the role of one particular miRNA found previously in literature or previous investigation.

## 3. Discussion

Despite the great interest in miRNAs in biomedical research, there are only few experimental and clinical studies on miRNAs in renal IRI and KT. These studies focus on the feasibility of measuring miRNAs and exploring their role in IRI. The clinical studies are characterized by their small sample size. Comparing clinical studies with IRI experiments in small animals is difficult. In animal experiments, IRI is induced by warm ischemia without transplantation. In clinical studies, kidneys underwent substantial cold ischemia, which may influence miRNA expression, and were subjected to alloreactivity in the recipient. Furthermore, animal experiments focus on IRI whilst clinical studies study biomarker potential for rejection or IF/TA.

Hitherto, no specific early biomarker is available for ischemic kidney damage after transplantation. In recent years it has been found that the kinetics of miRNAs in regulating mRNA translation is more complex than previously thought. Regulation succeeds through binding of a miRNA to the mRNA, but feedback loops are also involved as shown in TGF-*β*1 regulation in the kidney [46]. That and the fact that one single miRNA is able to suppress translation of multiple mRNA make it even more difficult to interpret miRNA expression levels alone without studying the effects on target pathways.

miRNAs have the potential to act as specific biomarkers, since it has been shown that miRNAs are dysregulated in disease states as found first in B-cell leukemia [47]. Unfortunately, the different studies revealed different miRNAs as a possible biomarker with no or little overlap between these studies. This may be the result of using different models, biomaterials, or different time points. An ideal biomarker is reproducible, with high specificity and sensitivity. When all reports are taken together, no single miRNA has been reproducibility identified as biomarker. For sensitivity, a reliable assay should be available. This is not yet the case. Even more, the lack of ready-to-use, reproducible assays could bias the results of the studies. When microRNAs found in microarrays are validated with PCR, only those microRNAs which give comparable expression levels are investigated further. This could result in a bias, where only those microRNAs will be examined further, which are validated through PCR. Future studies using RNA sequencing techniques which provide both qualitative and quantitative data could overcome these problems.

Because of their small size and stability, miRNAs could also be used as a therapeutic target by inactivating them using antagomirs or as a therapeutic agent as locked nucleic acids [48, 49]. Two reports studied differences in miRNA expression between untreated renal IRI and IRI ameliorated by known protective mechanisms, in an attempt to find miRNAs, which are involved in protection against IRI. mir-21 was found to be upregulated after IPC, and knockdown diminished the protective effect of IPC [19]. miR-21 thus contributes to the protective effect of IPC on renal IRI. miR-21 was also found to be expressed in tubular epithelial cells [17]. Knockdown resulted in an increased cell death. Nevertheless, overexpression of miR-21 did not result in increased cell survival after oxidative stress. Furthermore, inhibition of miR-21 is only detrimental in combination with IPC. Thus, although miR-21 is obviously involved in protection against IRI, it seems to be part of a more complex mechanism [50]. This mechanism needs to be unravelled to understand the interactions between miRNA-21 and other factors in renal IRI.

Although miRNAs have a great potential as biomarker, therapeutic target, and therapeutic agent in KT, the most important conclusion to be drawn at present is that miRNA research has not led to significant new insights into the pathophysiology of renal IRI, graft rejection, or tolerance and has failed to come up with clinically applicable biomarkers, therapeutic targets, or agents. This could be the result of differences in design of the studies, different tissues used to measure miRNA expression levels, different time points used, and different platforms to determine expression levels of miRNAs. Therefore, studies using more comparable aims, models, patient populations, and expression platforms are needed to determine if miRNAs are able to live up to their expectations in kidney transplantation.

## Figures and Tables

**Table 1:** Up- and downregulated miRNAs found in *in vivo* studies. miRNA−: downregulated miRNA, miRNA+: upregulated miRNA, MV: microvesicles, EPC: endothelial progenitor cells, and AmiR = antagomir.

Author	Species	Focus	Control	Tissue	Time point	MicroRNA−	MicroRNA+
Wei et al. [16], 2010	Mice	Dicer−/− in proximal tubules 30 min renal IRI	Wild type	Cortex	12 h	miR-18 miR-127 miR-135b miR-296 miR-322 miR-379 miR-487b miR-491	miR-17-3p miR-132 miR-207 miR-362 miR-489 miR-685 miR-687
					48 h	miR-324-3p miR-379 miR-455-3p	miR-7 miR-132 miR-362 miR-467 miR-486 miR-495 miR-668 miR-694

Godwin et al. [17], 2010	Mice	30 min renal IRI	Sham	Kidney	1-3-5-7-14-21-30 days	miR-187 miR-192 miR-194 miR-805	miR-20a miR-21 miR-146a miR-199a-3p miR-214

Xu et al. [19], 2012	Mice	15 min preconditioning before 30 min renal IRI	IRI/IRI + anti-miR-21	Kidney	4 h 24 h 4 days		miR-21

Jia et al. [28], 2013	Mice	Xenon preconditioning before 30 min renal IRI	IRI/IRI + anti-miR-21	Kidney	24 h		miR-21

Kaucsár et al. [24], 2013	Mice	20 and 30 min IRI	Sham	Kidney	24 h 3 days 4 days		miR-17-5 miR-21 miR-106

Saikumar et al. [18], 2012	Rat	30 min renal IRI	Sham	Kidney cortex	24 h		miR-21 miR-155 miR-18a

Cantaluppi et al. [20], 2012	Rat	MV EPC + 45 min renal IRI	Sham IRI IRI MV EPC (+RNAse/siRNA/AmiR126/296) IRI MV fibroblast	Kidney	2 days 7 days 180 days		miR-126 miR-296

**Table 2:** Overview of experiments using human (kidney) tissue. LTx = liver transplantation. IF/TA = interstitial fibrosis and tubular atrophy. STA = soluble transplant antigen. AMR = antibody-mediated rejection. DGF = delayed graft function.

Author	Group	Focus	Control	Tissue	Time point after transplantation	MicroRNA−	MicroRNA+
Sui et al. [9], 2008	Human live kidney donors	Acute rejection	Tumor nephrectomy	Cortex	Histologically confirmed acute rejection	miR-17-3p MM1 miR-197 MM2 miR-326 miR-330 MM1 miR-346 miR-483 miR-516 miR-524∗ miR-611 miR-654 miR-663	miR-125a MM1 miR-125a miR-320 miR-381 miR-602 miR-628 miR-629 miR-658

Anglicheau et al. [10], 2009	Human living + deceased donors	Acute rejection	Normal allograft	Biopsy	Variable	Let-7c miR-10a miR-10b miR-30a miR-30b miR-30c miR-30e-3p miR-32 miR-125a miR-200a	miR-142-3p miR-142-5p miR-146a miR-146b miR-155 miR-223 miR-342

Lorenzen et al. [11], 2011	Human living + deceased donors	Acute rejection	Normal allograft/urinary tract infection	Biopsy, urine	6 weeks 3 months 6 months	miR-10b miR-210	miR-10a

Scian et al. [13], 2011	Human deceased donors	IF/TA	Normal allograft	Biopsy, urine	>9 months	miR-107 miR-204 miR-211	miR-32 miR-142-3p

Ben-Dov et al. [12], 2012	Human living + deceased donors	IF/TA	Normal allograft	Biopsy, urine	Variable	miR-30 family	miR-21 miR-21∗ miR-142-3p miR-223 miR-506 miR-508 miR-509-3p miR-509-5p miR-514a

Danger et al. [15], 2012	Human living + deceased donors	STA	Operationally tolerant/healthy volunteers/LTx recipient	PBMCs	Variable	miR-106b miR-142-3p miR-450b-3p miR-876-3p	miR-98 miR-148b miR-324-5p miR-508-3p

Danger et al. [32], 2013	Human PBMC	Chronic AMR	Acute rejection/healthy volunteers/renal failure	PBMCs	Variable		miR-142-5p

Wilflingseder et al. [14], 2013	Human living + decreased donors	AMR	Normal allograft	Biopsy	Variable		Let-7i miR-21∗ miR-146b-5p miR-182 miR-663 miR-1228
		Acute rejection				Let-7b miR-23b miR-27b miR-30c-2∗ miR-99b miR-99b∗ miR-125a miR-125b-2∗ miR-138 miR-139-5p miR-181a miR-181b miR-193b miR-361-5p miR-424∗ miR-455 miR-502-3p miR-574-3p	miR-150 miR-155 miR-663a miR-638
		DGF					miR-17 miR-18a miR-20a miR-21∗ miR-106a miR-106b miR-182

Maluf [34], 2014	Human deceased donors	IF/TA	Normal allograft	Urine	Variable	miR-23b miR-30a miR-99a miR-125b miR-184 miR-193b miR-200b miR-203 miR-375 miR-513a-5p miR-575	Let-7f-2 miR-92a miR-106b miR-140a-3p miR-185 miR-345 miR-423-5p miR-425 miR-451 miR-486-5p

**Table 3:** Overview of *in vitro* experiments. PBMC = peripheral blood mononuclear cells. HREC = human renal epithelial cells. PTEC = proximal tubular epithelial cells. mTEC = murine tubular epithelial cells.

Author	Group	Focus	Control	Time point	MicroRNA−	MicroRNA+
Muratsu-Ikeda et al. [44], 2012	HK-2 cells	Hypoxia reoxygenation	Sham	3 h 10 h	miR-205	

Aguado-Fraile et al. [43], 2012	NRK52E cells HK-2 cells	Hypoxia reoxygenation	Sham			miR-127

Anglicheau et al. [10], 2009	PBMC	PHA stimulation	Sham		miR-223 Let-7c miR-142-5	miR-155
	HREC		Sham		miR30a-3p	

Godwin et al. [17], 2010	mTEC	Mineral oil	miR-21 KO miR-21++			miR-21

Xu et al. [19], 2012	HREC	Hypoxia	Normoxia			miR-21

Zell et al. [45], 2014	PTEC	Hypoxia	Normoxia		miR-124	
